# Knowledge about cardiovascular diseases in a first-level healthcare center in Lima, Peru

**DOI:** 10.17843/rpmesp.2024.413.13575

**Published:** 2024-08-28

**Authors:** César G. Lecarnaqué-Rojas, Javier I. Guerrero-Cueva, Otto Barnaby Guillén-López

**Affiliations:** 1 Alberto Hurtado School of Medicine, Universidad Peruana Cayetano Heredia, Lima, Peru. Universidad Peruana Cayetano Heredia Alberto Hurtado School of Medicine Universidad Peruana Cayetano Heredia Lima Peru; 2 Arzobispo Loayza National Hospital, Lima, Peru. Arzobispo Loayza National Hospital Lima Peru

**Keywords:** Knowledge, Heart Disease Risk Factors, Primary Health Care

## Abstract

This study aimed to determine the level of knowledge about cardiovascular diseases in people in a primary healthcare center (PHCC). A descriptive and cross-sectional study was carried out by surveying people who attended a PHCC in Lima, Peru. A score less than 6 was considered inadequate knowledge. A total of 400 people were surveyed, 66.3% were women and the mean age was 46.8 ± 16.2 years. The average score was 4.52 +/- 1.85. We found that 71% of those surveyed had an inadequate level of knowledge, regardless of age, gender or education level. Our findings show that the level of knowledge about risk factors and cardiovascular disease was inadequate in the primary care population. It is necessary to achieve proper specific education in cardiovascular risk factors in order to reduce the impact of these diseases.

## INTRODUCTION

Atherosclerotic cardiovascular diseases (CVD) are the leading cause of mortality worldwide ^1^. By 2018, ischemic cardiac and cerebrovascular events accounted for 11.3% of deaths in Peru, representing an increase of more than 200% compared to 2010 ^2^. Some modifiable cardiovascular risk factors that can be addressed in medical care are smoking, physical activity, dyslipidemias, obesity, diabetes mellitus (DM), and arterial hypertension (AHT) ^3^. In Peru, 24.6% of the population aged 15 years or older suffered from obesity, 21.7% from AHT, and 4.1% from DM in 2020 ^4^.

Given the pathophysiology of CVD, primary prevention is recommended ^5^. Therefore, adequate education and knowledge of risk factors is essential to prevent these diseases ^1^.

Studies from Northern Ireland and Kuwait have shown that the greater the knowledge of cardiovascular risk factors, the lower the prevalence of these diseases in the population ^6^^,^^7^. And a higher level of knowledge about these factors may also improve the adherence of patients with CVD to healthy lifestyles ^8^. In Peru, 53% of patients with AHT had little knowledge about their disease ^9^. However, these studies were conducted in more complex facilities, and only explored the knowledge of specific risk factors. Nevertheless, most preventive activities for these diseases are carried out at the first level of health care (FLHC) ^10^.

In our setting, there is a lack of studies on the knowledge of other CVD in the population who do not have these diseases. Therefore, this study aimed to determine the level of knowledge about cardiovascular diseases and some risk factors in persons attending a FLHC health center in Lima, Peru.

**KEY MESSAGES**

Motivation for the study. In Peru, the knowledge level of patients about their cardiovascular health is unknown at the first level of care, which receives 85% of the population, and where primary disease prevention activities are carried out.

Main findings. More than 70% of people had an inadequate level of knowledge about their own cardiovascular health.

Implications. Our results highlight the need to improve the education of people on cardiovascular health issues at the first level of care, taking into account that these diseases are increasingly frequent in the population.

## THE STUDY

We conducted a descriptive cross-sectional study with people who attended the I-3 Amakella Health Center (located in the district of San Martín de Porres in Lima, Peru), and their companions or family members. The sampling was carried out by convenience between September and October 2022.

The inclusion criteria were the following: persons of both sexes who attended the health care center for any reason, and their companions or family members, all aged 18 years or older. Pregnant women were excluded (as this would alter the results of blood pressure and abdominal circumference), as well as people with cognitive deficits or neurological sequelae that would make it impossible for them to complete the questionnaire, and people who did not speak Spanish.

Data was collected during interviews by using a questionnaire to assess knowledge of cardiovascular risk and disease. The questionnaire was validated in Spanish with patients treated in the community by Amariles *et al.*^11^, who authorized us to adapt their instrument. Our questionnaire was assessed by 8 physicians who are experts in treating patients with cardiovascular disease (2 cardiologists, 2 endocrinologists, and 4 internists). Subsequently, we carried a pilot study out in 10 persons with similar characteristics to those in this study on a single day in the same health center, in order to evaluate how the questions were understood. The instrument was validated for its application, and a coefficient of 0.89 Aiken’s V test was obtained; a Cronbach’s α of 0.93. This showed that the instrument had high reliability in these aspects.

The final questionnaire had 18 questions (8 evaluated demographic and anthropometric variables, and 10 evaluated the level of knowledge). Among the anthropometric variables, we included abdominal circumference, which was not categorized to define obesity; and body mass index (BMI), divided into four categories: obesity (BMI ≥ 30 kg/m^2^), overweight (BMI 25-29.9), normal weight (BMI 18.5-24.9) and underweight (BMI < 18.5). For older adults, BMI was categorized as follows: obese (BMI ≥ 32 kg/m^2^), overweight (BMI 28.0-31.9), normal (BMI 23.0-27.9), and underweight (BMI < 23.0).

Each correct answer was worth 1 point, and 0 points if it was wrong. The total score ranged from 0 to 10 points (“0” represented worse knowledge and “10” better knowledge). A score of “6” or more was classified as adequate knowledge ^11^, and categorized as poor knowledge (score < 3), fair (score between 3 and 5), good (score between 6 and 8), and excellent (score > 8).

After collecting and analyzing the information, it was transferred to a database in Microsoft Excel® 2020 and descriptive statistics were used to calculate frequencies and percentages, measures of central tendency and dispersion.

This study was approved by the Institutional Research Ethics Committee (CIEI) of the Universidad Peruana Cayetano Heredia (UPCH) on August 16, 2022 in document N° 358-31-22.

## FINDINGS

The questionnaire was applied to 400 people, and 265 (66.3%) were women. The average age was 46.8 + 16.2 years. The average BMI was 28.3 + 5.2 kg/m^2^. Thirty-one percent were obese, 34% were overweight, 31.25% had normal weight and only 3.75% were underweight. The average abdominal circumference was 94.9 + 13.2 cm (95.0 cm in women and 92.3 cm in men). Other characteristics of the participants are presented in Table 1.

**Table 1:** Demographic characteristics of people in the Amakella Health Center of San Martin de Porres in Lima, Peru.

Variable	Frequency	Percentage (%)
Sex		
Male	135	33.8
Female	265	66.2
Age group		
Young	76	19.0
Adult	214	53.5
Elderly	110	27.5
Level of education		
Illiterate	2	0.5
Completed primary school	22	5.5
Incomplete primary school	14	3.5
Completed Secondary school	122	30.5
Incomplete secondary school	53	13.2
Completed higher education	90	22.5
Incomplete higher education	97	25.3
Body mass index		
Low weight	15	3.75
Normal weight	125	31.25
Overweight	136	34.0
Obesity	124	31.0
Total	400	100.0

The average score was 4.52 + 1.85, and 71% of the participants obtained an inadequate score. Question 9 obtained the highest number of correct answers (76.5%) and was about knowing that smoking is a cardiovascular risk factor, while question 5 had the lowest number of correct answers (11.3%) and was about the adequate levels of total cholesterol in the blood (Table 2).

**Table 2:** Number of correct answers per question in people in the Amakella Health Center of San Martín de Porres in Lima, Peru.

Question number (*)	Subject	n	Percentage (%)
9	Relationship between smoking and cardiovascular disease	306	76.5
8	Relationship between diabetes and cardiovascular disease	297	74.3
2	What is the best type of exercise?	247	61.8
7	Relationship between triglycerides and cardiovascular disease	242	60.5
1	Perception of self-weight	179	44.8
10	Normal blood pressure values	147	36.8
6	HDL and LDL cholesterol implications	119	29.8
4	Risk of heart attack by sex	114	28.5
3	Risk of heart attack according to age	112	28.0
5	Normal total cholesterol values	45	11.3

(*) More details can be found in the supplementary material.

An inadequate level of knowledge was obtained in all educational levels. However, all those with excellent knowledge had secondary or higher education. We found that 77.6% of young people, 71.9% of adults,64.5% of older adults, 74.1% of men and 69.4% of women had inadequate knowledge. Most people had inadequate knowledge, regardless of their BMI (Table 3).

**Table 3:** Level of knowledge according to characteristics of the people in the Amakella Health Center of San Martín de Porres in Lima, Peru.

		Knowledge level									
		Poor		Fair		Good		Excellent		Total	
		n	%	n	%	n	%	n	%	n	%
Age groups											
	Young (18-29 years)	13	17.1	46	60.5	15	19.7	2	2.7	76	19.0
	Adult (30-59 years)	38	17.7	116	54.2	56	26.2	4	1.9	214	53.5
	Elderly (60 years and older)	15	13.6	56	50.9	36	32.7	3	2.8	110	27.5
Sex											
	Male	20	14.8	80	59.3	32	23.7	3	2.2	135	33.8
	Female	46	17.3	138	52.1	75	28.3	6	2.3	265	66.3
Level of education											
	Primary school (complete or incomplete)	2	5.3	6	68.4	10	26.3	0	0.0	38	9.5
	Completed Secondary school	9	16.9	33	62.3	10	18.9	1	1.9	53	13.3
	Incomplete secondary school	28	22.9	67	54.9	25	20.6	2	1.6	122	30.5
	Completed higher education	12	12.4	50	51.6	33	34.0	2	2.0	97	24.3
	Incomplete higher education	15	16.7	42	46.7	29	32.2	4	4.4	90	22.5
Body mass index											
	Low weight	1	6.7	10	66.7	3	20.0	1	6.6	15	3.75
	Normal weight	18	14.4	66	52.8	34	27.2	7	5.6	125	31.25
	Overweight	22	16	72	52.6	41	30.0	1	1.4	136	34.0
	Obesity	21	17	74	59.6	29	23.4	0	0	124	31.0
Total		62	15.5	222	55.5	107	26.7	9	2.3	400	100.0

## DISCUSSION

According to our results, 71% of respondents had inadequate knowledge of cardiovascular disease, with an average score of 4.52 out of a maximum of 10. This result is lower than that obtained by Amariles *et al*. ^12^, who reported an average score of 5.8, and that obtained in Colombia, which was 5.4 ^13^. In the Netherlands, knowledge was slightly better, with 42.3% of patients with inadequate perception of cardiovascular risk ^14^. These differences may be due to the fact that these studies included only patients with a diagnosed cardiovascular disease. This could have affected their knowledge of cardiovascular risk due to previous education of the patient regarding these diseases by their treating physician. Our study included persons who did not necessarily have a previous diagnosis of cardiovascular disease.

Regarding age, the majority of participants had an inadequate level of knowledge. This was similar to another study conducted in Lima, Peru, which reported that patients had a medium or low level of knowledge, independently of their age group ^9^. However, in our study, older adults had better knowledge, which contrasts with that of Spain, where the proportion of patients with good or excellent knowledge was higher in those under 60 years of age ^11^.

Regarding sex, almost 70% of women had inadequate knowledge. This is similar to what was found in another study in Lima, which showed that 53.5% of women had inadequate knowledge ^9^. In Venezuela, the highest percentage of men showed a low and intermediate level of knowledge, while approximately half of the women had an intermediate or high level of knowledge ^15^. These differences are not only due to cognitive or educational aspects intrinsic to sex, but possibly because more women than men were interviewed in our study.

According to the level of education, two thirds of the people who obtained an excellent score had a higher level of education, results similar to those obtained in Spain and the Netherlands ^12^^,^^14^. However, having a higher education would not necessarily imply having adequate knowledge, since most participants with higher education in our study did not have it.

Almost 80% of participants with obesity had inadequate knowledge, which was higher than in participants from the overweight or normal weight group. Another study in Peru reported that less knowledge was also found in obese people than in those who were overweight, especially for risk factors such as nutritional status and metabolic syndrome ^16^.

It is worth noting that 55% of people with obesity were wrong in question 1, which asked them to indicate their own nutritional status, which was also higher than those with overweight or normal weight. This could be explained by the fact that the definition of obesity may be subjective for each person and subject to different perceptions or social constructs ^17^.

On the other hand, just under 75% of people recognized diabetes as a cardiovascular risk factor, slightly more than in Venezuela or India ^15^^,^^18^. This difference could be explained by the increase in the prevalence of diabetes in recent years, which could increase knowledge of this disease, either because they suffer from it or know someone who does. Likewise, almost 80% of people recognized smoking as a cardiovascular risk factor. This is similar to data reported in Pakistan ^19^, and could be due to the influence of the media on smoking, which could result in a negative reinforcement of smoking over time ^20^.

Few people identified normal cholesterol values and blood pressure status. This was similar to what was found by a study carried out in Venezuela, which reported that less than half of the people identified elevated cholesterol and arterial hypertension as cardiovascular risk factors ^15^. This could be due to the fact that these diseases, being asymptomatic, may be poorly recognized as health problems or cardiovascular risk factors by the general population. On the other hand, the information in classic media about the prevention of these diseases may not be as widespread as smoking.

Our research had some limitations. One of the main limitations was the fact that the descriptive design of the study did not allow us to establish a cause-effect relationship or to generalize the results. Besides, there may have been information bias, due to problems in the interpretation of the questions by some people. Then, it was not possible to verify if the answers were reliable or if they were selected randomly. Finally, selection bias could have occurred due to the non-probabilistic sampling.

Nevertheless, our study has valuable aspects. It has public health implications, since we found people in primary care who did not have adequate knowledge of cardiovascular health. We also included not only patients with CVD, but also their companions, which broadened the possibility of capturing people who might never have been captured in a health care center before, since they did not have any medical condition at the time of the survey.

In conclusion, knowledge of cardiovascular disease and some of its risk factors was inadequate in the studied population, independently of gender, stage of life, and level of education. Knowledge of diabetes and smoking as cardiovascular risk factors was better in most cases. However, less than half of the respondents recognized overweight or obesity, normal blood pressure and cholesterol values.

Therefore, there is a need to ensure that patients recognize the risk factors for CVD and are motivated to adopt healthy lifestyles to prevent these diseases. Some educational interventions, not only in health care centers but also through mass media, could improve the general population’s knowledge of these diseases. Future studies with probabilistic, multicenter sampling, comparing knowledge between the population with and without CVD, can be carried out to better target such interventions.
