# Supplementary material for: Knowledge about cardiovascular diseases in a first-level healthcare center in Lima, Peru
Source: Rev Peru Med Exp Salud Publica. 2024 Aug 28;41(3):281–6. doi: 10.17843/rpmesp.2024.413.13575 (PMC11495947; doi:10.17843/rpmesp.2024.413.13575)
Supplement: Supplementary material. — Available in the electronic version of the RPMESP. [file rpmesp-41-03-13575-s001.docx]

**MATERIAL SUPLEMENTARIO**

**PREGUNTAS DE RIESGO Y ENFERMEDAD CARDIOVASCULAR**

**Marque una sola respuesta según lo que usted considere apropiado. Puede marcar “No sabe” si es que no conoce la respuesta.**

**1. Según su peso y su talla actual, usted considera que tiene o está:**

A. Peso bajo

B. Peso normal

C. Sobrepeso

D. Obesidad

E. No sabe

**2. Según lo que usted conoce de hacer ejercicio, ¿cuál de las siguientes situaciones es la mejor?**

A. Correr 45 min. dos veces a la semana

B. Caminar 45 min cuatro veces a la semana

C. Hacer ejercicio no genera beneficios para la salud

D. No sabe

**3. Entre dos hombres cuya única diferencia es que uno tiene 50 años y el otro 60 años, con respecto al riesgo de sufrir un infarto al corazón, usted piensa que:**

A. Tienen igual riesgo

B. El de 60 años tiene menor riesgo

C. El de 50 años tiene menor riesgo

D. No sabe

**4. Entre un hombre y una mujer que tienen los dos 55 años, con respecto al riesgo de sufrir un infarto al corazón, usted piensa que:**

A. Tienen igual riesgo

B. El hombre tiene menor riesgo

C. La mujer tiene menor riesgo

D. No sabe

**5. Se tiene a una persona con un valor de colesterol total de 167 mg/dL. Según este valor, usted cree que**:

A. Está dentro de lo normal

B. Está por encima de lo normal

C. Está por debajo de lo normal

D. No sabe

**6. Con respecto a los diferentes tipos de colesterol (HDL y LDL), usted piensa que:**

A. Tener ambos niveles altos es malo para la salud.

B. Tener ambos niveles altos es bueno para la salud

C. Hay un colesterol bueno (HDL) y otro malo (LDL)

D. No sabe

**7. Con respecto a los triglicéridos y las enfermedades cardiovasculares, usted piensa que:**

A. No tienen relación

B. Evitan su aparición

C. Favorecen su aparición

D. No sabe

**8. Con respecto a la diabetes y el desarrollo de enfermedades cardiovasculares, usted piensa que:**

A. La diabetes y las enfermedades cardiovasculares no tienen relación

B. El sufrir de diabetes favorece el desarrollo de enfermedades cardiovasculares

C. El sufrir de diabetes impide el desarrollo de enfermedades cardiovasculares

D. No sabe

**9. Entre dos pacientes hombres de 60 años que tienen hipertensión arterial hay uno que sí fuma y otro que no fuma. Con respecto al riesgo de sufrir una enfermedad al corazón usted piensa que:**

A. Los dos tienen el mismo riesgo por tener hipertensión arterial

B. El que sí fuma tiene menor riesgo

C. El que no fuma tiene menor riesgo

D. No sabe

**10. ¿Cuál es su presión arterial actual?:____/____. De acuerdo a su valor de presión arterial actual, usted considera que:**

A. Son normales o está controlada

B. Están un poco elevadas

C. Están muy elevadas

D. No sabe

Según el número de pregunta, las respuestas correctas son:

[Respuesta variable]*

1. B
2. C
3. C
4. A
5. C
6. C
7. B
8. C
9. [Respuesta variable]*

***** Estas respuestas se contrastan con la información médica correcta.

**Adaptado de:**

Amariles P, Pino-Marín D, Sabater-Hernández D, García-Jiménez E, Roig-Sánchez I, Faus MJ. Fiabilidad y validez externa de un cuestionario de conocimiento sobre riesgo y enfermedad cardiovascular en pacientes que acuden a farmacias comunitarias de España. Aten Primaria. 2016 Nov;48(9):586-595. doi: 10.1016/j.aprim.2016.01.005
