# Supplementary material for: Knowledge about cardiovascular diseases in a first-level healthcare center in Lima, Peru
Source: Rev Peru Med Exp Salud Publica. 2024 Aug 28;41(3):281–6. doi: 10.17843/rpmesp.2024.413.13575 (PMC11495947; doi:10.17843/rpmesp.2024.413.13575)
Supplement: Translation

# Translation: Conocimientos sobre enfermedades cardiovasculares en un establecimiento de primer nivel de atención en Lima, Perú

**Authors:** César G. Lecarnaqué-Rojas, Javier I. Guerrero-Cueva, Otto Barnaby Guillén-López

**Affiliations:** 1 Facultad de Medicina Alberto Hurtado, Universidad Peruana Cayetano Heredia, Lima, Perú.; 2 Hospital Nacional Arzobispo Loayza, Lima, Perú.

**Keywords:** Conocimiento, Atención Primaria de Salud, Factores de Riesgo de Enfermedad Cardiaca

## Abstract

El objetivo del estudio fue determinar el nivel de conocimiento sobre enfermedades cardiovasculares en personas en un centro de salud del primer nivel de atención en salud (PNAS). Se realizó un estudio descriptivo y transversal mediante una encuesta a personas que asistieron a un establecimiento de salud del PNAS en Lima, Perú. Un puntaje menor a 6 se consideró como conocimiento inadecuado. Se encuestó a 400 personas, 66,3% mujeres, de 46,8 ± 16,2 años. La nota promedio fue de 4,52 +/- 1,85. El 71% de los encuestados tuvo un nivel de conocimiento inadecuado, independientemente de la edad, género o grado de instrucción. Nuestros hallazgos muestran que el nivel de conocimiento sobre los factores de riesgo y la enfermedad cardiovascular resultó inadecuado en la población en atención primaria. Es necesario lograr una educación específica en factores de riesgo cardiovascular, para reducir el impacto de estas enfermedades.

## INTRODUCCIÓN

Las enfermedades cardiovasculares (ECV) ateroscleróticas son la primera causa de mortalidad a nivel mundial ^1^. Para el año 2018, los eventos isquémicos cardíacos y cerebrovasculares sumaban el 11,3% de muertes en Perú, representando un aumento de más del 200% respecto al 2010 ^2^. Algunos factores de riesgo cardiovascular modificables, abordables en la atención médica, son el tabaquismo, actividad física, dislipidemias, obesidad, diabetes mellitus (DM) e hipertensión arterial (HTA) ^3^. En Perú, 24,6% de la población de 15 años o más padecía de obesidad, el 21,7% de HTA, y el 4,1% de DM en el 2020 ^4^.

Dada la fisiopatología de las ECV, se recomienda la prevención primaria de estas ^5^. Entonces, resulta indispensable una educación y conocimiento adecuados sobre los factores de riesgo para prevenir estas enfermedades ^1^.

En Irlanda del Norte y Kuwait se evidenció que, a mayor conocimiento sobre factores de riesgo cardiovasculares, menor prevalencia de estas enfermedades en la población ^6^^,^^7^. Y un mayor nivel de conocimiento sobre esto puede mejorar también la adherencia de los pacientes con ECV a estilos de vida saludables ^8^. En Perú, 53% de pacientes con HTA presentaron un nivel bajo de conocimiento sobre su enfermedad ^9^. Sin embargo, estos estudios se realizaron en establecimientos de mayor complejidad, y sólo exploraron el conocimiento de factores de riesgo específicos. No obstante, la mayoría de actividades preventivas para estas enfermedades se realizan en el primer nivel de atención en salud (PNAS) ^10^.

En nuestro medio faltan estudios sobre el conocimiento de otras ECV en población que no tienen dichas enfermedades. Por ello, nuestro objetivo buscó determinar el nivel de conocimiento acerca de enfermedades cardiovasculares y algunos factores de riesgo en personas que acuden a un centro de salud del PNAS en Lima, Perú.

**MENSAJES CLAVE**

Motivación para realizar el estudio. En el Perú se desconoce el nivel de conocimiento de los pacientes acerca de su salud cardiovascular en el primer nivel de atención, donde se atiende el 85% de la población y se realizan actividades de prevención primaria de enfermedades.

Principales hallazgos. Más de 70% de personas tuvieron un nivel de conocimiento inadecuado sobre su salud cardiovascular.

Implicancias. Nuestros resultados ponen en evidencia la necesidad de mejorar la educación de las personas en temas de salud cardiovascular en el primer nivel de atención, teniendo en cuenta que estas enfermedades son cada vez más frecuentes en la población.

## EL ESTUDIO

Estudio descriptivo transversal realizado con personas que acudieron a atenderse en el Centro de Salud I-3 Amakella (localizado en el distrito de San Martín de Porres en Lima, Perú), y sus acompañantes o familiares. El muestreo fue realizado por conveniencia entre los meses de septiembre y octubre de 2022.

Los criterios de inclusión fueron: personas de ambos sexos que acudieron a atenderse a dicho establecimiento por cualquier motivo, y sus acompañantes o familiares, todos con edad de 18 años a más. Se excluyeron a gestantes (pues iba a alterar los resultados de presión arterial y circunferencia abdominal), personas con algún déficit cognitivo o secuela neurológica que les imposibilite realizar el cuestionario, y personas que no hablaban español.

La recolección de datos se realizó mediante entrevista, aplicando un cuestionario para evaluar conocimientos en torno a riesgo y enfermedad cardiovascular. Este fue validado en idioma español con pacientes atendidos en la comunidad por Amariles y col. ^11^, quien nos autorizó adaptar su instrumento. Para nuestro estudio, el cuestionario se sometió a un juicio de 8 médicos expertos en atender pacientes con enfermedades cardiovasculares (2 cardiólogos, 2 endocrinólogos y 4 médicos internistas). Posteriormente, se hizo un estudio piloto en 10 personas con características similares a las de este estudio en un día en el mismo centro de salud, en quienes se evaluó el entendimiento de las preguntas. El instrumento fue validado para su aplicación, y se obtuvo un coeficiente de 0.89 la prueba V de Aiken; un α de Cronbach de 0,93. Esto demostró que el instrumento tuvo alta fiabilidad en estos aspectos.

El cuestionario final tuvo 18 preguntas (8 evaluaron variables demográficas y antropométricas, y 10, el nivel de conocimiento). Dentro las variables antropométricas, se determinó la circunferencia abdominal, la cual no se categorizó para definir obesidad; y el índice de masa corporal (IMC), del cual se obtuvo 4 categorías: obesidad (IMC ≥ 30 kg/m^2^), sobrepeso (IMC 25-29,9), peso normal (IMC 18,5-24,9) y bajo peso (IMC < 18,5). Para adultos mayores, el IMC se categorizó de la siguiente manera: obesidad (IMC ≥ 32 kg/m2), sobrepeso (IMC 28,0-31,9), normal (IMC 23,0-27,9), y bajo peso (IMC < 23,0).

Cada respuesta correcta valió 1 punto, y 0 puntos si fue errónea. El puntaje total osciló entre 0 y 10 puntos (“0” representó un peor conocimiento y “10” un mejor conocimiento). Un puntaje de “6” o más se clasificó como conocimiento adecuado ^11^, y se categorizó como conocimiento deficiente (puntaje < 3), regular (puntaje entre 3 y 5), bueno (puntaje entre 6 y 8) y excelente (puntaje > 8).

Luego de la recopilación y análisis de la información, se trasladó a una base de datos en Microsoft Excel® 2020 y se utilizó estadística descriptiva para calcular frecuencias y porcentajes, medidas de tendencia central y de dispersión.

Este estudio fue aprobado por el Comité Institucional de Ética en Investigación (CIEI) de la Universidad Peruana Cayetano Heredia (UPCH) el 16 de agosto de 2022 en el documento N° 358-31-22.

## HALLAZGOS

El cuestionario fue aplicado a 400 personas, y 265 (66,3%) fueron mujeres. El promedio de edad fue de 46,8 + 16,2 años. El IMC promedio fue 28,3 + 5,2 kg/m2. Un 31% de personas tuvo obesidad, 34% sobrepeso, 31,25% peso normal y solo 3,75% delgadez. La circunferencia abdominal promedio fue 94,9 + 13,2 cm (en mujeres fue 95,0 cm y en varones 92,3 cm). Las demás características de los participantes se presentan en la Tabla 1.

**Tabla 1:** Características demográficas de las personas en el Centro de Salud Amakella de San Martín de Porres en Lima, Perú.

Variable		Frecuencia	Porcentaje (%)
Sexo			
	Masculino	135	33,8
	Femenino	265	66,2
Grupo etáreo			
	Joven	76	19,0
	Adulto	214	53,5
	Adulto mayor	110	27,5
Grado de instrucción			
	Iletrado	2	0,5
	Primaria completa	22	5,5
	Primaria incompleta	14	3,5
	Secundaria completa	122	30,5
	Secundaria incompleta	53	13,2
	Superior completa	90	22,5
	Superior incompleta	97	25,3
Índice de masa corporal			
	Bajo peso	15	3,75
	Normal	125	31,25
	Sobrepeso	136	34,0
	Obesidad	124	31,0
	Total	400	100,0

La nota promedio obtenida fue de 4,52 + 1,85. El 71% de personas obtuvo un puntaje inadecuado. La pregunta 9 obtuvo la mayor cantidad de aciertos (76,5%) e indagaba sobre el conocimiento acerca del tabaquismo como factor de riesgo cardiovascular, mientras que la pregunta 5 obtuvo menor cantidad de aciertos (11,3%) y trataba sobre los niveles adecuados de colesterol total en sangre (Tabla 2).

**Tabla 2:** Cantidad de aciertos por pregunta en personas en el Centro de Salud Amakella de San Martín de Porres en Lima, Perú.

Número de pregunta (*)	Tema	n	Porcentaje (%)
9	Relación del tabaco y enfermedad cardiovascular	306	76,5
8	Relación de diabetes y enfermedad cardiovascular	297	74,3
2	¿Cuál es el mejor tipo de ejercicio?	247	61,8
7	Relación de triglicéridos y enfermedad cardiovascular	242	60,5
1	Percepción del peso propio	179	44,8
10	Valores normales de la presión arterial	147	36,8
6	Implicancias de HDL y LDL colesterol	119	29,8
4	Riesgo de infarto al corazón según sexo	114	28,5
3	Riesgo de infarto al corazón según edad	112	28,0
5	Valores normales de colesterol total	45	11,3

(*) Puede verse más detalles en el material suplementario

Un nivel de conocimiento inadecuado se obtuvo en todos los grados de instrucción. No obstante, todos los que tuvieron un conocimiento excelente tenían grado de instrucción secundaria o superior. Según el grupo etario y el sexo, 77,6% de jóvenes, 71,9% de adultos y 64,5% de adultos mayores; y, 74,1% de varones y 69,4% de mujeres tuvieron conocimiento inadecuado, respectivamente. La mayor parte de personas tuvieron un conocimiento inadecuado, cualesquiera que fuese su IMC (Tabla 3).

**Tabla 3:** Nivel de conocimiento según características de las personas en el Centro de Salud Amakella de San Martín de Porres en Lima, Perú.

		Nivel de conocimiento									
		Deficiente		Regular		Bueno		Excelente		Total	
		n	%	n	%	n	%	n	%	n	%
Grupos etarios											
	Joven (18-29 años)	13	17,1	46	60,5	15	19,7	2	2,7	76	19,0
	Adulto (30-59 años)	38	17,7	116	54,2	56	26,2	4	1,9	214	53,5
	Adulto mayor (60 años a más)	15	13,6	56	50,9	36	32,7	3	2,8	110	27,5
Sexo											
	Masculino	20	14,8	80	59,3	32	23,7	3	2,2	135	33,8
	Femenino	46	17,3	138	52,1	75	28,3	6	2,3	265	66,3
Grado de instrucción											
	Primaria (completa o incompleta)	2	5,3	6	68,4	10	26,3	0	0,0	38	9,5
	Secundaria incompleta	9	16,9	33	62,3	10	18,9	1	1,9	53	13,3
	Secundaria completa	28	22,9	67	54,9	25	20,6	2	1,6	122	30,5
	Superior incompleta	12	12,4	50	51,6	33	34,0	2	2,0	97	24,3
	Superior completa	15	16,7	42	46,7	29	32,2	4	4,4	90	22,5
Índice de masa corporal											
	Bajo peso	1	6,7	10	66,7	3	20,0	1	6,6	15	3,75
	Normal	18	14,4	66	52,8	34	27,2	7	5,6	125	31,25
	Sobrepeso	22	16	72	52,6	41	30,0	1	1,4	136	34,0
	Obesidad	21	17	74	59,6	29	23,4	0	0	124	31,0
Total		62	15,5	222	55,5	107	26,7	9	2,3	400	100,0

## DISCUSIÓN

Según nuestros resultados, 71% de entrevistados tuvo un conocimiento inadecuado sobre enfermedad cardiovascular, con un puntaje promedio de 4,52 de un máximo de 10. Este resultado es menor al obtenido por Amariles *et. al.*^12^, cuya nota promedio fue de 5,8; y al obtenido en Colombia donde fue 5,4 ^13^. En Países Bajos, el conocimiento fue un poco mejor con 42,3% de pacientes con percepción inadecuada del riesgo cardiovascular ^14^. Estas diferencias se deberían a que en estos estudios se incluyó solo pacientes con alguna enfermedad cardiovascular diagnosticada. Esto pudo afectar su conocimiento sobre el riesgo cardiovascular por educación previa del paciente respecto a estas enfermedades por parte de su médico tratante. En nuestro estudio se incluyeron personas que no necesariamente tenían un diagnóstico previo de alguna enfermedad cardiovascular.

En relación con la edad, la mayoría obtuvo un nivel inadecuado de conocimiento. Esto fue similar a otro estudio realizado en Lima Perú donde los pacientes tenían un nivel de conocimiento medio o bajo, independientemente de su grupo etario ^9^. No obstante, en nuestro estudio los adultos mayores tuvieron un mejor conocimiento, lo cual contrasta con el de España, donde la proporción de pacientes con conocimiento bueno o excelente fue mayor en los menores de 60 años ^11^. 

En relación al sexo, casi el 70% de las mujeres tuvo conocimiento inadecuado. Esto se asemeja a lo encontrado en otro estudio de Lima, donde 53,5% de mujeres tuvieron conocimiento inadecuado ^9^. En Venezuela, el mayor porcentaje de los hombres mostró un nivel de conocimiento bajo e intermedio, mientras que aproximadamente la mitad de las mujeres presentó un nivel intermedio o alto de conocimiento ^15^. Estas diferencias no se deberían solo a aspectos cognitivos o educativos intrínsecos al sexo, sino posiblemente porque se entrevistaron más mujeres que varones en nuestro estudio.

Según el grado de instrucción, las dos terceras partes de personas que obtuvieron un puntaje excelente tenían un grado de instrucción superior, resultados similares a los obtenidos en España y Países Bajos ^12^^,^^14^. Sin embargo, tener una educación superior no implicaría necesariamente tener un conocimiento adecuado, pues la mayor parte de personas con educación superior en nuestro estudio no lo tuvieron.

Entre las personas con obesidad, casi 80% tuvieron un conocimiento inadecuado, lo cual fue mayor que el grupo con sobrepeso o peso normal. En otro estudio en Perú, se encontró igualmente un menor conocimiento en obesos que en los que tenían sobrepeso, en especial para factores de riesgo como el estado nutricional y el síndrome metabólico ^16^.

Algo llamativo fue que 55% de personas con obesidad se equivocaron en la pregunta 1, que pedía indicar su propio estado nutricional, lo que fue mayor también que aquellos con sobrepeso o peso normal. Esto podría explicarse porque la definición de obesidad puede ser subjetiva para cada persona y estar sujeta a percepciones o constructos sociales distintos ^17^.

Por otro lado, poco menos de 75% de personas reconocieron a la diabetes como factor de riesgo cardiovascular, un poco más que en Venezuela o la India ^15^^,^^18^. Esta diferencia podría explicarse por el aumento de la prevalencia de diabetes en los últimos años, que podría aumentar el conocimiento sobre esta enfermedad, sea por padecerla o conocer alguien que la padezca. Asimismo, casi 80% de personas reconocieron al tabaquismo como un factor de riesgo cardiovascular. Esto guarda similitud con datos reportados en Pakistán ^(^^19^, y podría deberse a la influencia de los medios de comunicación sobre el tabaquismo, que pudo resultar en un reforzamiento negativo para fumar en el tiempo ^20^.

Pocas personas identificaron los valores normales de colesterol y el estado de su presión arterial. Esto fue similar a lo hallado en Venezuela, donde también se vio que menos de la mitad de personas identificaron al colesterol elevado y a la hipertensión arterial como factores de riesgo cardiovascular ^15^. Esto podría deberse a que estas enfermedades, siendo asintomáticas, podrían ser poco reconocidas como problemas de salud o de riesgo cardiovascular por la población general. Por otro lado, la información en los medios de comunicación clásicos acerca de la prevención de estas enfermedades puede no ser tan mediática como con el tabaquismo.

Nuestra investigación tuvo algunas limitaciones. Primero, el diseño descriptivo del estudio no permite establecer una relación causa-efecto o generalización de resultados. Segundo, pudo existir sesgo de información, por problemas de interpretación de las preguntas por algunas personas. Tercero, no se podía verificar si las respuestas eran confiables o si se marcaron al azar. Cuarto, pudo ocurrir un sesgo de selección por usar muestreo no probabilístico.

No obstante, nuestro estudio tiene aspectos valiosos. Primero, tiene implicancias de salud pública, pues se encontraron personas en atención primaria que no tuvieron un adecuado conocimiento sobre salud cardiovascular. Segundo, incluimos no solo pacientes con ECV, sino a sus acompañantes, lo cual ampliaba la posibilidad de captar personas que quizás nunca antes serían captadas en un establecimiento de salud, al no tener patologías en el momento de la encuesta.

En conclusión, el conocimiento sobre enfermedad cardiovascular y algunos de sus factores de riesgo resultó inadecuado en la población estudiada, independientemente de su género, etapa de vida y grado de instrucción. El conocimiento de la diabetes y el tabaco como factores de riesgo cardiovascular fue mejor en la mayoría de casos. Sin embargo, el reconocimiento del sobrepeso u obesidad y valores normales de presión arterial y colesterol fueron conocidos por menos de la mitad de los encuestados.

Existe entonces la necesidad de lograr que los pacientes reconozcan los factores de riesgo de ECV y sean motivados a adoptar estilos de vida saludables para prevenir estas enfermedades. Mediante algunas intervenciones educativas, no solo en centros de atención de salud sino en medios de comunicación masiva, se podría mejorar el conocimiento de la población general sobre estas enfermedades. Estudios futuros con muestreo probabilístico, multicéntricos, comparando el conocimiento entre población con enfermedad cardiovascular y sin ella, pueden ser realizados para dirigir mejor dichas intervenciones.
